# Gut microbiota and health-related quality of life in paediatric inherited metabolic disorders: a narrative review and conceptual framework

**DOI:** 10.3389/fmicb.2026.1928657

**Published:** 2026-09-28

**Authors:** John Sieh Dumbuya, Yuanlong Li, Bo He, Guangxu Li, Yuting Liu, Youfeng Zhong, Shimei Huang, Xiuling Chen

**Affiliations:** 1 Department of Paediatrics, The First Affiliated Hospital of Hainan University, Haikou People’s Hospital, Haikou, Hainan, China; 2 Department of Paediatrics, Hainan Women and Children's Medical Centre, Haikou, Hainan, China

**Keywords:** gut microbiota, health-related quality of life, inherited metabolic disorders, narrative review, phenylketonuria, probiotics, short-chain fatty acids

## Abstract

Inherited metabolic disorders (IMDs) comprise more than 1,400 rare genetic conditions affecting paediatric patients worldwide. Despite advances in newborn screening and dietary management, many children experience chronic complications, gastrointestinal symptoms, and reduced health-related quality of life (HRQoL). Emerging evidence—predominantly from phenylketonuria (PKU)—suggests that gut microbiota may influence metabolic homeostasis and patient-centred outcomes in affected children. Because of substantial methodological heterogeneity and the rarity of individual disorders, we elected a narrative review format to synthesise current evidence rather than a formal quantitative synthesis. Electronic searches were performed in PubMed, Embase, and Web of Science from inception to February 2026. We synthesise current evidence regarding gut microbiota composition in major paediatric IMDs, including PKU, organic acidemias, glycogen storage diseases, lysosomal storage disorders, and mitochondrial diseases. For PKU, controlled studies report reduced microbial diversity and depletion of butyrate-producing taxa associated with long-term dietary treatment. For the remaining disorders, direct paediatric microbiome data are sparse or absent, and proposed links rely on mechanistic extrapolation from non-IMD populations. We present the gut microbiota–metabolism–quality of life axis as a conceptual framework that integrates microbial metabolites, intestinal barrier function, immune signalling, and patient-reported outcomes, while emphasising that direct causal evidence linking microbiota alterations to HRQoL in paediatric IMDs is currently lacking. Microbiota-directed interventions remain preliminary. Future multicentre longitudinal studies integrating microbiome profiling, multi-omics technologies, and validated patient-reported outcomes are needed to clarify causal mechanisms and therapeutic potential. Importantly, the available evidence is heterogeneous: direct paediatric microbiome evidence is strongest for PKU, whereas evidence for other IMDs is sparse and often observational, preclinical, or extrapolated from non-IMD populations. The proposed microbiota–metabolism–HRQoL axis is therefore presented as a hypothesis-generating framework rather than an established causal pathway.

## Graphical abstract

**Figure:**
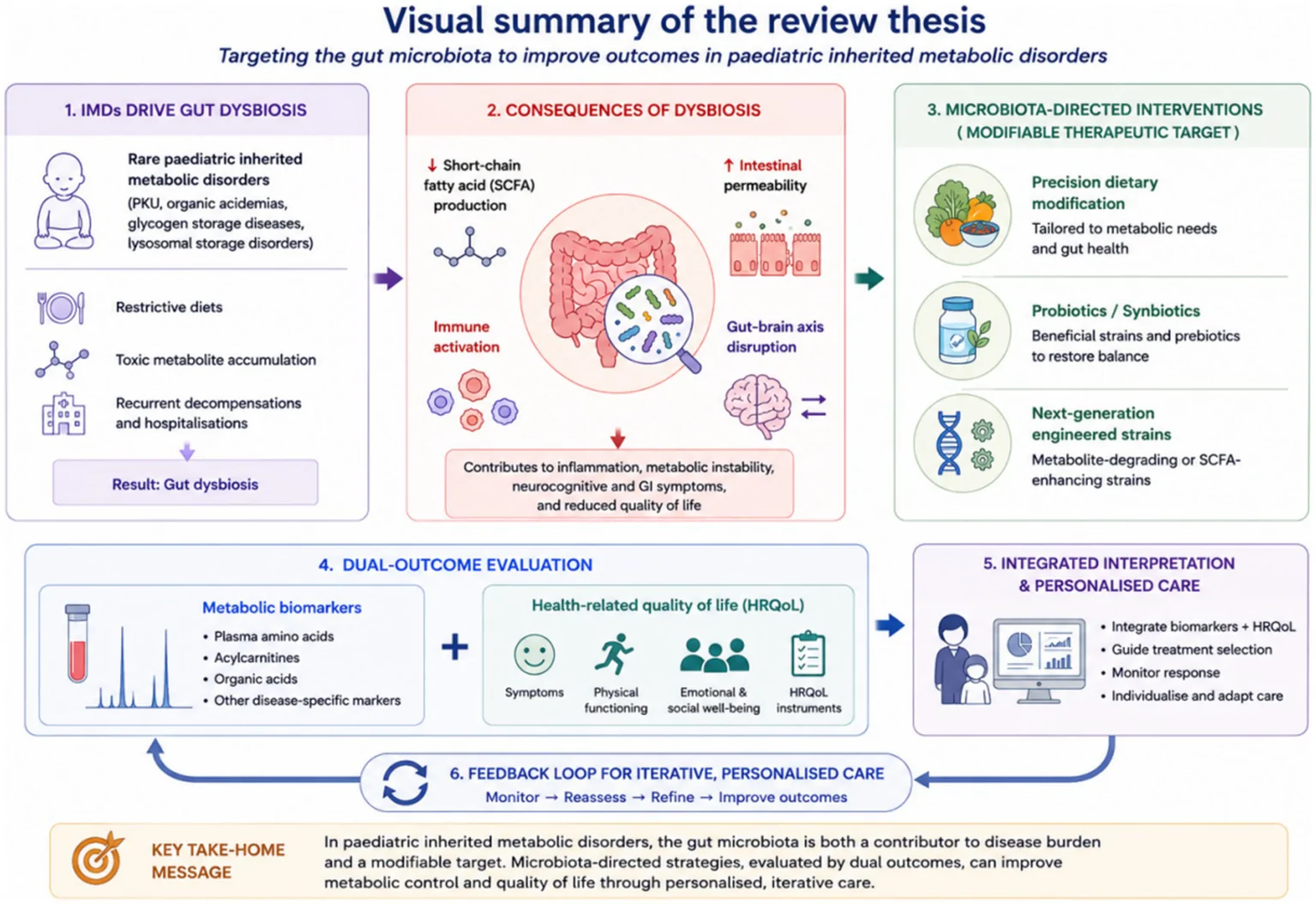
Visual summary of the review thesis. Rare paediatric inherited metabolic disorders (PKU, organic acidemias, glycogen storage diseases, lysosomal storage disorders) drive characteristic gut dysbiosis through restrictive diets, toxic metabolite accumulation, and recurrent decompensations. Dysbiosis manifests as reduced short-chain fatty acid (SCFA) production, increased intestinal permeability, immune activation, and gut–brain axis disruption. Microbiota-directed interventions—including precision dietary modification, probiotics/synbiotics, and next-generation engineered strains—offer a modifiable therapeutic target. The review advocates for dual-outcome evaluation integrating metabolic biomarkers with health-related quality of life measures, creating a feedback loop for iterative, personalised care.

## 1 Introduction

Inherited metabolic disorders (IMDs) comprise more than 1,400 rare genetic conditions caused by defects in metabolic pathways, disrupting the synthesis, degradation, transport, or utilisation of essential biomolecules (Ferreira et al., 2021; van Karnebeek, 2018). Collectively, IMDs are not uncommon, with birth prevalence ranging from approximately 1 in 800 to 1 in 2,500 live births (Ferreira et al., 2021; Pampols, 2010). Manifestations include acute metabolic crises, growth disturbances, and lifelong treatment burden (Baumgartner et al., 2014; Ferreira et al., 2021). Advances in newborn screening and dietary management have improved survival for many affected children (Bouchereau et al., 2024; van Karnebeek, 2018). Nevertheless, patients experience chronic complications and reduced health-related quality of life (HRQoL) despite optimised management (Eminoglu et al., 2013; Pascoal et al., 2018). The gastrointestinal tract harbours a complex microbial ecosystem functioning as a metabolic, immunological, and neuroendocrine organ (Cani, 2018; Cryan et al., 2019). Gut microbiota regulate barrier integrity, immune homeostasis, and gut–brain communication through metabolites, including short-chain fatty acids (SCFAs) (Cryan et al., 2019; Koh et al., 2016; Strandwitz, 2018). Interest has emerged regarding gut microbiota in IMDs. Restrictive diets and recurrent decompensations influence microbial ecology (Guerrero et al., 2019; Salazar et al., 2023). Microbial metabolites may influence permeability, immune activation, and neurobehavioural pathways (Cani et al., 2007; Cryan et al., 2019). However, the evidence remains fragmented and heavily centred on PKU.

Among IMDs, the strongest and most replicated microbiome-related evidence currently exists for PKU, where controlled studies have reported alterations in microbial diversity and reductions in butyrate-producing bacteria associated with long-term dietary treatment (Mancilla et al., 2021; Ostrowska et al., 2024). Evidence for microbiota alterations in organic acidemias, glycogen storage diseases, lysosomal storage disorders, and mitochondrial diseases remains considerably more limited, consisting largely of case reports, animal models, or theoretical extrapolation from non-IMD populations (Bordugo et al., 2021; Kirby et al., 2019). Recognition of patient-reported outcomes in rare diseases has grown. Children with IMDs experience psychosocial stress, dietary restrictions, gastrointestinal symptoms, neurocognitive difficulties, and caregiver burden extending beyond traditional biochemical indicators of disease control (Bösch et al., 2021; Pascoal et al., 2018; Zeltner et al., 2014). Accordingly, findings from PKU are not assumed to be representative of other paediatric IMDs, and the evidence is interpreted separately by disease category.

To our knowledge, this is the first narrative review to explicitly integrate microbiome evidence with HRQoL and patient-reported outcomes across multiple paediatric IMD categories. While previous reviews by Kirby et al. (2019), Mancilla et al. (2021), and Ostrowska et al. (2024) have described microbial alterations in PKU and other IMDs; none have systematically examined how these microbiota changes may intersect with patient-centred outcomes, including gastrointestinal symptom burden, neurobehavioural functioning, and caregiver impact. The conceptual framework presented here—the gut microbiota–metabolism–quality of life axis—represents a novel synthesis that bridges microbial metabolites, intestinal barrier function, immune signalling, and patient-reported outcomes, without implying that these domains form an established causal pathway. This framework is presented not as an established causal pathway, but as a hypothesis-generating structure to guide future research design and to emphasise the clinical relevance of microbiome science beyond descriptive microbial profiling.

The objectives of this narrative review are therefore fourfold: (1) to summarise current evidence regarding gut microbiota composition and function in paediatric IMDs, with explicit attention to the strength of evidence for each disorder; (2) to critically examine proposed mechanisms linking microbial alterations to metabolic dysfunction, gastrointestinal symptoms, and neurobehavioural outcomes, presenting these as a conceptual framework rather than established causal pathways; (3) to evaluate the current evidence supporting microbiota-directed therapeutic interventions, emphasising their experimental status; and (4) to explore implications for HRQoL and patient-reported outcomes, acknowledging that direct microbiota–QoL links in IMDs remain unproven. Throughout the review, direct paediatric IMD evidence is distinguished from mechanistic evidence derived from non-IMD populations, and causal interpretations are avoided where only associative evidence is available.

## 2 Literature search strategy

This narrative review critically evaluates evidence on alterations in the gut microbiota in paediatric inherited metabolic disorders (IMDs) and their associations with health-related quality of life. We selected a narrative format because the extreme rarity of individual IMDs, heterogeneity of study designs, and the absence of uniform microbiome methodologies preclude meaningful quantitative synthesis or meta-analysis, as required by systematic scoping reviews. Instead, we aimed to map the breadth of evidence, identify knowledge gaps, and generate hypotheses for future research. The review process was designed to maximise transparency while recognising that it was not intended to meet all methodological requirements of a PRISMA systematic review.

Electronic searches were conducted in PubMed, Embase, and Web of Science from database inception to February 2026. The search strategy combined controlled vocabulary (MeSH/Emtree) and free-text terms grouped into four conceptual domains: (i) gut microbiota (e.g., “microbiota,” “dysbiosis,” “gut flora,” “16S rRNA”); (ii) inherited metabolic diseases (e.g., “inborn errors of metabolism,” “phenylketonuria,” “organic acidemia,” “glycogen storage disease,” “lysosomal storage disease,” “mitochondrial disease”); (iii) paediatric population (e.g., “child,” “paediatric,” “infant,” “adolescent”); and (iv) outcomes and interventions (e.g., “quality of life,” “probiotics,” “prebiotics,” “faecal microbiota transplantation”). No language restrictions were applied.

The electronic searches retrieved approximately 3,400 records across the three databases. After automated and manual deduplication, approximately 2,850 unique records remained for title and abstract screening. Of these, 215 full-text articles were assessed for relevance. Ultimately, 108 studies were included in this narrative synthesis based on their direct contribution to one or more of the following domains: (a) gut microbiota composition or function in paediatric IMDs; (b) validated HRQoL or patient-reported outcome assessment in paediatric IMDs; or (c) mechanistic pathways (SCFA metabolism, barrier function, gut–brain signalling) with explicit relevance to the IMD population. Reasons for exclusion at the full-text stage were recorded and included: (i) exclusive adult populations without paediatric-specific data or subgroup analysis; (ii) non-IMD metabolic conditions (e.g., type 2 diabetes mellitus, obesity without an underlying inborn error of metabolism); (iii) conference abstracts without available peer-reviewed full text; (iv) studies lacking primary microbiome data, validated HRQoL instruments, or direct mechanistic relevance; and (v) non-English language articles for which translatable text could not be obtained. A simplified study flow diagram is provided as Supplementary Figure S1. Screening was performed by title/abstract, followed by full-text assessment against the predefined eligibility domains. The study-selection process is summarised in Supplementary Figure S1. Because the review is narrative, the numerical counts are provided for transparency rather than as a PRISMA-compliant systematic review flow.

Given IMD rarity, available literature consists primarily of small observational studies and preclinical investigations. Findings are presented according to evidence strength, distinguishing established clinical observations from mechanistic hypotheses and emerging therapeutic concepts. For synthesis, evidence was qualitatively stratified as controlled/clinical observational evidence, uncontrolled or case-based clinical evidence, experimental/animal evidence, and mechanistic extrapolation from non-IMD populations.

## 3 Gut microbiota in health and relevance to metabolic homeostasis

The gut microbiota develops after birth and undergoes dynamic changes through infancy and childhood before adolescent stabilisation (Bäckhed et al., 2015; Yatsunenko et al., 2012). Healthy commensals exhibit functional redundancy, maintaining metabolic function despite fluctuations in microbial composition (Faith et al., 2013). In the context of IMDs, three host–microbiota interaction domains are particularly relevant. First, bacterial fermentation of dietary fibre yields SCFAs—acetate, propionate, and butyrate—which serve as colonocyte fuel, maintain tight-junction integrity, promote regulatory T-cell differentiation, and modulate energy expenditure via G-protein-coupled receptors (Koh et al., 2016; Peng et al., 2009; Round and Mazmanian, 2009; Smith et al., 2013; van de Wouw et al., 2018). In IMDs characterised by protein or fibre restriction (e.g., PKU, organic acidemias), reduced substrate availability for fermentation may directly compromise SCFA production and barrier function (Mancilla et al., 2021; Ostrowska et al., 2024). Second, microbiota modify bile acid pools and signalling. Primary bile acids undergo microbial biotransformation into secondary bile acids, interacting with FXR and TGR5 to influence glucose homeostasis and lipid metabolism (De Vadder et al., 2014; Pathak et al., 2018; C. Thomas et al., 2009; Wahlström et al., 2016). Whether these interactions are altered in paediatric IMDs remains largely unexplored. Third, the intestinal microbiota shapes immune development and mucosal homeostasis through bidirectional microbe–immune crosstalk (Round and Mazmanian, 2009). Disruption contributes to altered permeability and chronic low-grade inflammation implicated in metabolic syndrome (Cani et al., 2007; Viggiano et al., 2015). Similar mechanisms in IMDs are hypothesised but unproven. The gut–brain axis involves neural, endocrine, immune, and metabolic pathways (Carabotti et al., 2015). Microbial metabolites may influence neurotransmitter synthesis and neuroinflammation (Erny et al., 2015; Macfabe, 2012; Strandwitz, 2018), yet causal relationships between microbiota alterations and neurocognitive outcomes in paediatric IMDs remain unestablished. In interpreting these mechanisms, dietary substrate availability, medical formula composition, antibiotic exposure, feeding modality, age, and disease severity are considered potential modifiers rather than assuming that microbial differences are disease-specific.

Microbial composition alone may not capture functionality. Metagenomics and multi-omics reveal that metabolic activity provides more clinically relevant information than taxonomic profiles (Franzosa et al., 2014; Zierer et al., 2018). This is particularly important in IMDs, where metabolic abnormalities and dietary interventions alter microbial function independent of abundance. Variability in sample collection, sequencing platforms, and bioinformatic analyses complicates comparisons and limits reproducibility (Costea et al., 2017). These challenges are pronounced in rare IMDs, where small samples and heterogeneity preclude firm conclusions. These methodological differences also limit the strength of cross-study comparisons; consequently, taxonomic differences are interpreted as descriptive findings unless supported by functional or longitudinal evidence.

## 4 Gut microbiota alterations in major paediatric IMDs

### 4.1 Phenylketonuria (PKU)

Phenylketonuria is the most extensively studied IMD in microbiome research. PKU results from a deficiency of phenylalanine hydroxylase, leading to impaired conversion of phenylalanine to tyrosine and accumulation of phenylalanine in blood and tissues (Blau et al., 2010). Lifelong treatment relies primarily on dietary restriction of natural protein combined with phenylalanine-free medical formulas, although pharmacological approaches such as sapropterin and pegvaliase have expanded therapeutic options (Burton et al., 2010; Scala et al., 2024). Several controlled observational studies have demonstrated reduced microbial diversity and depletion of butyrate-producing taxa in individuals with PKU. Decreased abundance of *Faecalibacterium prausnitzii, Roseburia, Coprococcus*, and other beneficial Firmicutes has been reported, accompanied by relative enrichment of Streptococcus, *Lactobacillales*, and taxa associated with altered carbohydrate metabolism (Mancilla et al., 2021; Ostrowska et al., 2024). These findings are biologically plausible given the substantial dietary differences between individuals with PKU and healthy controls, particularly lower intake of natural protein and fibre-rich foods. Multiple controlled studies with consistent findings support reduced microbial diversity and depletion of butyrate-producing taxa in PKU (Table 1). Although PKU provides the most consistent paediatric IMD microbiome evidence, these observations remain largely observational and are strongly intertwined with long-term dietary therapy; they should therefore not be extrapolated directly to other IMDs.

**Table 1:** Strength of evidence for gut microbiota alterations in major paediatric inherited metabolic disorders.

Disorder	Evidence grade	Key supporting studies	Type of evidence	Main limitations	Confidence in microbiota–HRQoL link
Phenylketonuria (PKU)	High	Mancilla et al. (2021), Ostrowska et al. (2024), Pinheiro de Oliveira et al. (2016)	Multiple controlled observational studies with 16S rRNA sequencing; cross-sectional and preliminary longitudinal designs	Cross-sectional designs preclude causality; dietary confounding is not fully controlled; adult data predominate	Moderate: microbial alterations are replicated, but direct causal links to HRQoL remain unproven
Organic Acidemias (MMA, PA)	Low	Bordugo et al. (2021), Kirby et al. (2019)	Case reports; small uncontrolled series; mechanistic hypotheses and animal models	No controlled paediatric cohort studies; antibiotic exposure confounding; dietary restriction not standardised	Very Low: biological plausibility exists but no direct evidence links microbiota to HRQoL in this population
Glycogen Storage Diseases	Low	Hannibal et al. (2020), Koopman et al. (2012)	Small cohort studies; metabolic profiling with inferred microbial associations	Extremely limited direct microbiome data; confounding by cornstarch therapy and simple-carbohydrate load; single-centre studies	Very Low: no studies have concurrently assessed microbiota and HRQoL in paediatric GSD
Lysosomal Storage Disorders	Very Low	De Vadder et al. (2014), Koopman et al. (2012), Thomas et al. (2021)	Theoretical extrapolation from GI physiology; no disease-specific microbiome studies	Absence of direct paediatric microbiome investigations; GI symptoms attributed primarily to mechanical organomegaly rather than microbial alterations	Very Low: purely hypothetical at present
Mitochondrial Disorders	Very Low	Alula et al. (2023), Haque et al. (2024), Juárez-Fernández et al. (2023)	Experimental models; mechanistic hypotheses extrapolated from non-IMD metabolic disorders	No direct human paediatric microbiome studies; extreme heterogeneity of mitochondrial phenotypes; frequent antibiotic exposure and enteral feeding confounding	Very Low: biological plausibility from non-IMD models, but no direct evidence in paediatric mitochondrial disease

PKU, Phenylketonuria; MMA, methylmalonic acidemia; PA, Propionic acidemia; GSD, Glycogen storage disease; LSD, Lysosomal storage disorder; HRQoL, Health-related quality of life. Evidence grades: High = multiple controlled studies with consistent findings and replication; Moderate = at least one controlled study or multiple small observational studies with consistent directional findings; Low = case reports, small uncontrolled series, or strong mechanistic plausibility without direct paediatric data; Very Low = theoretical extrapolation from non-IMD populations or absence of disease-specific studies.

Functional consequences remain incompletely understood. Investigations suggest reductions in SCFA production, particularly butyrate, which may influence intestinal barrier integrity and immune regulation, although this remains an inferred biological consequence rather than a demonstrated clinical effect in paediatric PKU (Mancilla et al., 2021; Ostrowska et al., 2024; Pinheiro de Oliveira et al., 2016). Investigators have proposed links between dysbiosis, gastrointestinal symptoms, neurocognitive outcomes, and metabolic control (van Vliet et al., 2022; Zhang et al., 2025; Ziadlou and MacDonald, 2026). However, current evidence is predominantly cross-sectional and cannot establish causality. Neurocognitive complications remain an important concern in PKU despite early diagnosis and treatment. Executive dysfunction, attention deficits, anxiety, and reduced psychosocial well-being have been reported even among individuals with relatively good metabolic control (Jahja et al., 2014; Remor et al., 2024). Although microbiota-mediated gut–brain interactions have been proposed as potential contributors, direct evidence linking specific microbial changes to neurocognitive outcomes in PKU remains limited and should be considered hypothesis-generating.

### 4.2 Organic acidemias

Organic acidemias, including methylmalonic acidemia (MMA) and propionic acidemia (PA), are characterised by enzymatic defects that impair metabolism of specific amino acids, resulting in accumulation of toxic organic acids and recurrent metabolic decompensation (Baumgartner et al., 2014). Several factors may predispose affected patients to microbiota disruption, including severe protein restriction, recurrent hospitalisations, frequent antibiotic exposure, gastrointestinal dysmotility, and chronic metabolic instability (Baumgartner et al., 2014; Salazar et al., 2023). However, direct evidence describing gut microbial composition in paediatric organic acidemias remains sparse; no controlled paediatric cohort studies have been published to date. The most detailed human evidence comes from isolated case reports and small observational studies. For example, Bordugo and colleagues described altered microbial diversity and reduced abundance of beneficial taxa in an infant with PA before probiotic supplementation (Bordugo et al., 2021). Experimental and theoretical models suggest that altered intestinal pH, accumulation of toxic metabolites, and repeated antimicrobial exposure may contribute to dysbiosis (Kirby et al., 2019). The relationship between propionic acid metabolism and microbial ecology is particularly complex: propionate is both a microbial metabolite and a pathologic metabolite in PA, creating interactions that remain poorly understood (Bordugo et al., 2021; Huang et al., 2021). In maple syrup urine disease (MSUD), elevated faecal and plasma branched-chain amino acids create a unique metabolic niche that may select for BCAA-utilising bacterial species while suppressing standard commensals, further altering the microbial landscape (Bordugo et al., 2021); however, this remains theoretical. Direct paediatric microbiome data are limited to case reports and small observational studies; no controlled cohort studies have been published to date (Table 1). The current evidence should therefore be regarded as low-certainty and hypothesis-generating, rather than as evidence of a reproducible disease-specific dysbiosis.

### 4.3 Glycogen storage diseases

Glycogen storage diseases (GSDs) comprise a heterogeneous group of disorders characterised by abnormalities in glycogen synthesis or degradation (Wraith et al., 2008). Nutritional management frequently involves uncooked cornstarch therapy, continuous feeding regimens, and specialised dietary interventions that substantially alter substrate availability within the gastrointestinal tract. Patients with GSD type I exhibit reduced microbial diversity and altered abundance of Bacteroides and Firmicutes compared to healthy peers, effects attributed to the simple-carbohydrate load and insufficient complex fibre intake (Hannibal et al., 2020; Koopman et al., 2012). The reliance on simple carbohydrates rather than complex fibres reduces substrate for butyrate production, potentially compromising colonic health and barrier function (Faith et al., 2013; Viggiano et al., 2015). Additionally, hepatomegaly and portal hypertension in advanced disease may alter splanchnic circulation and intestinal permeability, further modifying the microbial ecosystem (Jones et al., 2009; Peng et al., 2009). Nevertheless, direct microbiome investigations in paediatric GSD populations remain extremely limited; existing data derive from small cohorts and require replication. Existing data derive from small cohorts and require replication; direct microbiome investigations in paediatric GSD populations remain extremely limited (Table 1). The available GSD microbiome evidence is therefore insufficient to separate the effects of the underlying disorder from those of cornstarch exposure, continuous feeding, fibre intake, and other treatment-related factors.

### 4.4 Lysosomal storage disorders

Lysosomal storage disorders (LSDs), including mucopolysaccharidoses (MPS), arise from deficiencies of lysosomal enzymes leading to progressive accumulation of undegraded substrates within multiple tissues (Wraith et al., 2008). These abnormalities may plausibly affect microbial composition through changes in gastrointestinal transit and nutrient availability, but direct investigations of gut microbiota in paediatric LSD populations are largely absent. Existing evidence consists primarily of mechanistic hypotheses extrapolated from gastrointestinal physiology rather than disease-specific microbiome studies. The expanded use of enzyme replacement therapy has improved somatic outcomes, yet GI symptoms remain prevalent; whether these reflect underlying microbial alterations rather than purely mechanical organomegaly is currently unknown (De Vadder et al., 2014; Koopman et al., 2012; Thomas et al., 2021). Direct investigations of gut microbiota in paediatric LSD populations are largely absent; existing evidence consists primarily of mechanistic hypotheses (Table 1). No disease-specific microbiome–HRQoL relationship can currently be inferred from the available paediatric LSD literature.

### 4.5 Mitochondrial disorders

Mitochondrial diseases are genetically heterogeneous disorders characterised by impaired oxidative phosphorylation and cellular energy production (Koopman et al., 2012). Gastrointestinal manifestations are common and may include dysmotility, feeding difficulties, chronic constipation, and intestinal pseudo-obstruction. Emerging evidence from non-IMD models suggests potential bidirectional interactions between host mitochondrial function and gut microbial metabolism (Alula et al., 2023; Haque et al., 2024; Juárez-Fernández et al., 2023). Microbial metabolites, particularly SCFAs, can influence mitochondrial bioenergetics, while mitochondrial dysfunction may alter intestinal barrier integrity and immune responses (Koh et al., 2016; Koopman et al., 2012). In humans, chronic acidosis, autonomic neuropathy, and frequent antibiotic exposure compound these effects, creating a profoundly altered intestinal milieu (Haque et al., 2024) (Table 2). Despite considerable biological plausibility, direct microbiome studies in paediatric mitochondrial disease remain scarce. Most proposed mechanisms are derived from experimental systems or extrapolated from common metabolic disorders. Despite biological plausibility, direct microbiome studies in paediatric mitochondrial disease remain scarce; most proposed mechanisms are derived from experimental systems or extrapolated from non-IMD populations (Table 1). Accordingly, the mitochondrial disease evidence is currently best classified as mechanistic or extrapolated evidence rather than established paediatric clinical evidence.

**Table 2:** Characteristic gut microbiota alterations in major paediatric inherited metabolic disorders.

Disorder	Key microbial alterations	SCFA profile	Primary drivers of dysbiosis	Clinical correlates
Phenylketonuria (PKU)	Reduced *Faecalibacterium prausnitzii, Roseburia, Coprococcus,* and *Barnesiella*; enrichment of *Streptococcus*, *Lactobacillales*, and *Eubacterium dolichum*; reduced *Firmicutes/Bacteroidetes* ratio in some cohorts	Markedly decreased total SCFAs, especially butyrate; reduced propionate and acetate	Lifelong restriction of natural protein and fibre; reliance on phenylalanine-free medical formulas low in complex carbohydrates; reduced aromatic amino acid substrate for commensal metabolism	Constipation, abdominal discomfort, increased intestinal permeability, neuropsychiatric comorbidities (anxiety, executive dysfunction), and possible increased risk of overweight/insulin resistance in adolescence
Organic Acidemias (MMA, PA)	Reduced microbial alpha-diversity; depletion of *Prevotella, Bacteroidetes*, and butyrate-producing taxa; expansion of facultative anaerobes	Decreased butyrate and acetate; potentially altered propionate levels (host-derived vs. microbial indistinguishable)	Severe protein restriction; recurrent metabolic decompensations and acidosis altering intestinal pH; frequent antibiotic exposure during acute crises; gut dysmotility from autonomic dysfunction	Severe feeding intolerance, recurrent vomiting, chronic constipation, failure to thrive, basal ganglia injury, and developmental delay
Maple Syrup Urine Disease (MSUD)	Depletion of standard commensals; selection for BCAA-utilising species; altered Firmicutes and Actinobacteria abundance	Reduced butyrate and altered branched-chain fatty acid metabolites	Extreme leucine, isoleucine, and valine restriction; accumulation of toxic BCAA derivatives; intermittent catabolic stress	Feeding difficulties, encephalopathy during decompensation, neurocognitive impairment, poor growth
Glycogen Storage Disease (Type I/III)	Reduced overall diversity; altered Bacteroides and Firmicutes ratios; depletion of fibre-fermenting species	Decreased butyrate due to low fibre intake; possible increase in simple sugar-fermenting taxa	High simple-carbohydrate, low-fibre diet (uncooked cornstarch/continuous glucose); hepatomegaly and portal hypertension altering splanchnic circulation	Constipation, diarrhoea, hepatic adenoma risk, lactic acidosis, long-term colonic dysmotility
Lysosomal Storage Disorders (MPS, Gaucher)	Systemic inflammation-associated dysbiosis hypothesised; reduced anti-inflammatory commensals hypothesised; possible expansion of proteolytic species	Inconsistent data; likely reduced butyrate in patients with chronic diarrhoea	Chronic systemic inflammation; substrate accumulation in tissues; enzyme replacement therapy altering immune milieu; frequent hospitalisations	Chronic diarrhoea or constipation, abdominal pain, hepatosplenomegaly, progressive multisystem involvement
Mitochondrial Disorders	Altered composition in mouse models; reduced SCFA producers hypothesised; potential expansion of oxidative-stress-resistant taxa	Reduced butyrate and propionate hypothesised	Impaired intestinal epithelial energy metabolism; altered motility from autonomic neuropathy; chronic acidosis; frequent antibiotic use	Severe GI dysmotility, constipation, feeding intolerance, failure to thrive, progressive neurological decline

BCAA, Branched-chain amino acid; GSD, Glycogen storage disease; LSD, Lysosomal storage disorder; MPS, Mucopolysaccharidosis; MMA, Methylmalonic acidemia; PA, Propionic acidemia; MSUD, Maple syrup urine disease; SCFA, Short-chain fatty acid.

## 5 Mechanisms linking dysbiosis to clinical outcomes and quality of life

Although multiple biological pathways have been proposed to explain interactions between gut microbiota and host physiology, direct mechanistic evidence in paediatric IMDs remains limited. Much of the current understanding derives from studies in obesity, inflammatory bowel disease, neurological disorders, and experimental animal models (Cani, 2018; Cryan et al., 2019; Koh et al., 2016; Strandwitz, 2018). Consequently, the mechanisms discussed below should be interpreted within the context of varying levels of evidence. While several pathways are well established in general microbiome research, their specific contribution to disease manifestations and HRQoL in paediatric IMDs remains incompletely defined. For clarity, mechanisms supported by experimental or non-IMD studies are presented as biologically plausible pathways; they should not be interpreted as demonstrated mechanisms of HRQoL impairment in paediatric IMDs.

Nevertheless, accumulating evidence suggests that alterations in microbial metabolites, intestinal barrier function, immune regulation, and gut–brain communication may represent biologically plausible links between dysbiosis and clinically relevant outcomes observed in children with IMDs, but the relative contribution of microbiota versus disease severity, diet, medication exposure, and treatment burden remains unknown (Guerrero et al., 2019; Salazar et al., 2023).

### 5.1 SCFA deficiency, barrier dysfunction, and immune dysregulation

Short-chain fatty acids (SCFAs), principally acetate, propionate, and butyrate, are among the best-characterised microbial metabolites influencing host physiology. Generated through bacterial fermentation of dietary fibre and other complex carbohydrates, SCFAs contribute to epithelial energy metabolism, maintenance of intestinal barrier integrity, immune regulation, and neuroendocrine signalling (Koh et al., 2016; Peng et al., 2009; Smith et al., 2013). Among SCFAs, butyrate serves as the primary energy substrate for colonocytes and promotes tight-junction assembly through activation of AMP-activated protein kinase and related signalling pathways (Peng et al., 2009; Round and Mazmanian, 2009). Experimental studies have also demonstrated that SCFAs influence regulatory T-cell differentiation and mucosal immune homeostasis (Smith et al., 2013). Several studies in PKU have reported reductions in butyrate-producing taxa and lower faecal SCFA concentrations compared with healthy controls (Mancilla et al., 2021; Ostrowska et al., 2024; Pinheiro de Oliveira et al., 2016). Similar alterations have been hypothesised in other IMDs, although direct evidence remains sparse. Consequently, it is biologically plausible that reduced SCFA availability may contribute to gastrointestinal symptoms frequently reported in IMDs, including constipation, abdominal discomfort, and altered bowel habits (Guerrero et al., 2019; Salazar et al., 2023). However, direct causal relationships between SCFA deficiency and clinical outcomes in paediatric IMDs have not yet been demonstrated. Importantly, reduced gastrointestinal well-being represents a recognised contributor to impaired HRQoL among children with IMDs and their families (Bösch et al., 2021; Eminoglu et al., 2013). Future studies integrating microbial metabolomics with patient-reported outcomes may help clarify whether SCFA alterations contribute meaningfully to these patient-centred outcomes. Importantly, the directionality of these associations is uncertain because dietary restriction, formula composition, antibiotic exposure, and gastrointestinal disease may themselves alter SCFA-producing communities.

The intestinal barrier plays a central role in maintaining homeostasis between luminal microorganisms and host immune responses. Disruption of this barrier may permit translocation of microbial products, including lipopolysaccharide (LPS), into systemic circulation, potentially promoting chronic inflammatory responses (Cani et al., 2007; Round and Mazmanian, 2009; Viggiano et al., 2015). Evidence linking dysbiosis to increased intestinal permeability is well established in several gastrointestinal and metabolic disorders (Peng et al., 2009; Round and Mazmanian, 2009). However, direct assessments of intestinal barrier integrity in paediatric IMDs remain limited. Reduced abundance of SCFA-producing organisms observed in PKU and other metabolic disorders has prompted hypotheses that impaired epithelial barrier function may occur in these populations (Mancilla et al., 2021; Ostrowska et al., 2024). Should barrier dysfunction occur, subsequent activation of Toll-like receptor 4 (TLR4) signalling and innate immune pathways could theoretically contribute to systemic inflammation, gastrointestinal symptoms, and disease burden (Cani et al., 2007). Nevertheless, direct evidence demonstrating increased intestinal permeability or endotoxaemia in paediatric IMDs is currently insufficient. Thus, increased permeability should not be inferred from microbial composition alone, and barrier-related mechanisms remain investigational in paediatric IMDs.

Given that chronic symptoms, recurrent hospitalisations, and treatment burden represent major determinants of HRQoL impairment in IMDs, future studies evaluating intestinal permeability biomarkers alongside clinical and patient-reported outcomes may provide important insights into disease mechanisms.

### 5.2 Gut–brain axis and neurobehavioural outcomes

Neurological complications represent a major source of morbidity in many IMDs. Cognitive impairment, executive dysfunction, anxiety, depression, behavioural disturbances, and reduced psychosocial functioning have been reported across multiple disease categories, particularly intoxication-type disorders such as PKU, MMA, and PA (Jahja et al., 2014; Zeltner et al., 2014). The gut–brain axis provides a conceptual framework through which microbial communities may influence neurological outcomes. Experimental studies in non-IMD models suggest that microbial metabolites can influence neurotransmitter synthesis, vagal signalling, microglial maturation, and neuroimmune regulation (Erny et al., 2015; Macfabe, 2012; Strandwitz, 2018). SCFAs have been implicated in the modulation of neuroinflammation and neuronal function (Erny et al., 2015). In animal models, propionic acid exposure has been associated with neurobehavioural alterations, generating interest in microbiota-mediated mechanisms relevant to organic acidemias (El-Ansary et al., 2013). Most of these observations originate from non-IMD experimental systems; their relevance to individual paediatric IMDs, and particularly to HRQoL, remains uncertain.

It is critical to emphasise that no interventional study in paediatric IMDs has demonstrated that modifying the gut microbiota improves neurocognitive or neurobehavioural outcomes. The gut–brain axis in IMDs remains a plausible but unproven mechanistic hypothesis.

### 5.3 Health-related quality of life and the gut microbiota

#### 5.3.1 Health-related quality of life in paediatric IMDs

IMDs are increasingly recognised as chronic conditions in which treatment success extends beyond biochemical control to include functional status, psychosocial wellbeing, and HRQoL. Advances in newborn screening, dietary management, enzyme replacement therapies, and disease-specific treatments have substantially improved survival; however, many children and families continue to experience persistent disease burden related to dietary restrictions, gastrointestinal symptoms, neurocognitive challenges, treatment complexity, and uncertainty regarding long-term outcomes (Bilder et al., 2016; Landolt et al., 2002; Zeltner et al., 2014).

HRQoL impairment in paediatric IMDs is heterogeneous across disease categories and instrument domains. For PKU—the most extensively studied disorder—total PedsQL Generic Core scores are consistently reported to be 5–15 points below healthy population norms, even among individuals with satisfactory metabolic control (Bilder et al., 2016; Jahja et al., 2014; Landolt et al., 2002). This “PKU paradox” reflects a disconnect between biochemical control and patient-centred outcomes. Subdomain analyses reveal that emotional functioning (anxiety, depressive symptoms) and school functioning (cognitive fatigue, executive dysfunction) are disproportionately affected, whereas physical functioning scores often approximate population norms in well-treated cohorts (Jahja et al., 2014; Remor et al., 2024).

In organic acidemias (MMA and PA), the HRQoL profile differs markedly. Total PedsQL scores frequently fall >20 points below healthy norms, with physical functioning domains particularly compromised due to recurrent metabolic crises, hospitalisations, and feeding intolerance (Bösch et al., 2021; Zeltner et al., 2014). The PedsQL Family Impact Module demonstrates elevated caregiver burden in these disorders, with “worry” and “daily activity disruption” subscores among the highest reported across chronic paediatric conditions (Pascoal et al., 2018). In glycogen storage diseases, physical functioning limitations predominate, driven by hypoglycaemia-related exercise intolerance and the burden of continuous feeding regimens; school functioning is often disrupted by the need for frequent daytime and nocturnal feeds (Kishnani et al., 2014). Lysosomal storage disorders present a progressive physical decline profile, with pain and physical health domains deteriorating over time; emotional functioning may remain relatively preserved in early stages, but declines as disease visibility increases (Jones et al., 2009; Wraith et al., 2008).

Cross-cutting subdomain patterns indicate that social functioning is relatively preserved in intoxication-type disorders managed by dietary restriction (PKU, MSUD) but severely compromised in progressive storage disorders with visible phenotypes (MPS). Caregiver-reported outcomes consistently exceed patient self-reported impairment across most IMDs, suggesting that family quality of life represents an independent therapeutic target (Pascoal et al., 2018; Shaji Thomas et al., 2021). These subdomain-specific patterns are clinically relevant because microbiota-directed interventions may differentially affect physical versus emotional versus social domains; future studies should therefore power analyses for subdomain effects rather than relying solely on total HRQoL scores.

The impact of IMDs extends beyond affected children and frequently involves substantial caregiver burden. Lifelong dietary management, emergency treatment protocols, frequent medical appointments, financial pressures, and concerns regarding disease progression may adversely affect parental well-being and family functioning (Pascoal et al., 2018; Zeltner et al., 2014). The PedsQL Family Impact Module and related instruments have demonstrated significant psychosocial burden among caregivers of children with metabolic disorders (Pascoal et al., 2018). These challenges may be exacerbated by gastrointestinal symptoms, feeding difficulties, and behavioural problems, which often require intensive daily management. Although direct evidence is lacking, interventions that successfully improve gastrointestinal well-being and reduce treatment burden could theoretically generate secondary benefits for family quality of life. This concept provides additional rationale for evaluating patient-reported and caregiver-reported outcomes in future microbiota-directed intervention studies.

Patient-reported outcome measures (PROMs), including the Pediatric Quality of Life Inventory (PedsQL), Child Health Questionnaire (CHQ), disease-specific IMD quality-of-life instruments, and gastrointestinal symptom scales, have increasingly been incorporated into IMD research (Pascoal et al., 2018; Ravens-Sieberer et al., 2005; Varni et al., 2007) (Table 3). These tools provide important information that is not captured by traditional biochemical markers alone. However, implementation remains inconsistent, and few studies have integrated PROMs with biological measures such as microbiome composition or microbial metabolite profiles. Instrument choice should account for developmental stage and informant: self-report is preferable when developmentally appropriate, whereas caregiver-proxy measures provide complementary information in younger or cognitively impaired children. Generic instruments such as PedsQL and EQ-5D-Y permit broader comparisons, while disease-specific tools and GI symptom measures may capture IMD-specific treatment burden that generic scales can miss.

**Table 3:** Summary of patient-reported outcome measures used in paediatric inherited metabolic disorders.

Instrument	Target age	Domains assessed	Disorders validated In	Sensitivity to change	Advantages	Limitations	MCID /Responsiveness notes
PedsQL Generic Core Scales	2–18 years (parent-proxy 2–4 yr.; child self-report 5–18 yr)	Physical, emotional, social, and school functioning	PKU, MSUD, MMA, PA, GSD, MPS, broad IMD cohorts	Moderate to high; responsive to metabolic control and dietary modifications	Extensive normative data; well-validated across cultures; brief administration	May lack sensitivity to IMD-specific concerns (e.g., dietary burden, crisis anxiety); ceiling effects in mild phenotypes	~4.5 points considered clinically meaningful for chronic conditions; subdomain changes of 4–6 points are often reported in IMD intervention studies
PedsQL Family Impact Module	Parents of children 2–18 years	Parents’ physical/emotional functioning, family daily activities, family relationships, and worry about the child	PKU, MMA, PA, GSD	High; sensitive to caregiver burden during crises and hospitalisations	Captures family-level impact; complements child-reported outcomes	Parent-proxy only; no child self-report version; burden varies by family structure	No IMD-specific MCID established; 5-point changes often correlate with clinically significant stress indicators
CHQ-PF50 / CHQ-CF87	Parent form 5–18 years; child form 10–18 years	Physical functioning, role/social limitations, bodily pain, behaviour, mental health, self-esteem, health perceptions, family activities/cohesion, change in health	PKU, GSD, LSDs, mixed IMD populations	Moderate; useful for comparison to healthy norms and chronic disease benchmarks	Comprehensive coverage of physical and psychosocial health; established chronic disease benchmarks	Lengthy administration; complex scoring algorithms; limited recent use in IMDs	MCID varies by domain (physical summary ~3 points, psychosocial summary ~5 points); less responsive to short-term dietary changes
WHO-5 Well-Being Index	Adolescents and adults (≥13 years)	Positive mood, vitality, general interest in daily activities	Adult PKU cohorts; limited paediatric data	Moderate; psychological well-being screening rather than comprehensive HRQoL	Ultra-brief (5 items); freely available; strong psychometric properties in adolescents	Narrow focus on positive well-being only; does not assess physical functioning, school, or social domains; limited paediatric IMD validation	10% score change (or ~5 raw points) is considered clinically relevant in depression screening contexts
EQ-5D-Y	8–18 years	Mobility, self-care, usual activities, pain/discomfort, anxiety/depression	Broad rare disease cohorts, including some IMDs	Low to moderate in IMDs; better suited for economic evaluation and utility assessment	Utility-based scoring enables QALY calculation; standardised across diseases	Ceiling effect in children with mild–moderate disease; limited sensitivity to IMD-specific issues; cognitive demands for the youngest ages	No established MCID in paediatric IMDs; primarily used for health-economic modelling rather than clinical trial endpoints
IMD-QoL Tool	Adolescents and adults	Disease-specific concerns: dietary burden, crisis anxiety, treatment adherence, social limitations	PKU, MSUD, MMA, PA (European Delphi consensus)	High for disease-specific interventions; limited by cross-language validation availability	Captures IMD-specific concerns missed by generic instruments; Delphi consensus content validity	Limited to European validation contexts; not widely translated; restricted age range	Delphi-derived thresholds; no empirically validated MCID; most useful for within-disorder comparisons
GSRS (Modified)	Adolescents/adults; parent-proxy for younger children	Abdominal pain, reflux, diarrhoea, constipation, indigestion	Organic acidemias, GSD, MPS	High for GI-targeted interventions; relevant for microbiota-directed trials	GI symptom specificity directly relevant to microbiome hypotheses; brief	Limited paediatric validation; symptom overlap with non-GI disease manifestations; parent-proxy reliability uncertain in young children	No established MCID in IMD populations; responsive to laxative and dietary fibre interventions in other paediatric GI disorders

PedsQL, Pediatric Quality of Life Inventory; CHQ, Child Health Questionnaire; EQ-5D-Y, EuroQol Five-Dimension Youth Version; IMD-QoL, Inherited Metabolic Diseases Quality of Life; GSRS, Gastrointestinal Symptom Rating Scale; PKU, Phenylketonuria; MSUD, Maple syrup urine disease; MMA, Methylmalonic acidemia; PA, Propionic acidemia; GSD, Glycogen storage disease; MPS, Mucopolysaccharidosis; LSD, Lysosomal storage disorder; HRQoL, Health-related quality of life; GI, Gastrointestinal.

#### 5.3.2 Potential links between gut microbiota and HRQoL

The biological plausibility of a relationship between gut microbiota and HRQoL arises from several interconnected pathways involving gastrointestinal function, immune regulation, metabolism, and the gut–brain axis. In healthy populations, microbial metabolites such as SCFAs, bile acid derivatives, and tryptophan metabolites contribute to intestinal homeostasis and influence signalling pathways involved in inflammation, neurodevelopment, and behaviour (Cryan et al., 2019; Koh et al., 2016; Strandwitz, 2018). In paediatric IMDs, several disease-related factors may alter these microbiota-mediated pathways. Restrictive therapeutic diets, reduced dietary fibre intake, specialised medical formulas, recurrent metabolic decompensations, and antibiotic exposure may influence microbial composition and function (Kirby et al., 2019; Mancilla et al., 2021; Ostrowska et al., 2024). These alterations could theoretically contribute to gastrointestinal symptoms, altered immune regulation, and neurobehavioural manifestations that affect daily functioning and quality of life. These pathways, therefore, provide a rationale for investigation, not evidence that microbiota alterations are independent determinants of HRQoL.

However, direct clinical evidence connecting microbiota alterations with HRQoL outcomes in children with IMDs remains extremely limited. Most existing studies have evaluated either microbiome composition or HRQoL independently, without integrating both domains within the same cohort. Therefore, while microbiota-related mechanisms provide a plausible biological framework, current evidence does not support the conclusion that dysbiosis directly causes impaired HRQoL. At present, no paediatric IMD study establishes a direct microbiota–HRQoL association with sufficient longitudinal or interventional evidence to support causality.

#### 5.3.3 Gastrointestinal symptoms as a potential mediator

Gastrointestinal symptoms represent one of the most clinically relevant potential links between microbiota alterations and quality of life in IMDs. Constipation, abdominal discomfort, feeding intolerance, vomiting, and altered bowel habits are frequently reported across several IMDs, including PKU, organic acidemias, GSDs, and lysosomal storage disorders (Guerrero et al., 2019; Salazar et al., 2023). In PKU, reduced fibre intake and reliance on phenylalanine-free medical foods may influence intestinal microbial composition and contribute to constipation and gastrointestinal discomfort (Mancilla et al., 2021; Ostrowska et al., 2024). Similar concerns have been raised in organic acidemias, where protein restriction, metabolic instability, and gastrointestinal dysmotility may influence both microbial ecology and symptom burden (Baumgartner et al., 2014; Costea et al., 2017). However, whether microbiota alterations are a primary contributor to gastrointestinal symptoms or a secondary consequence of dietary therapy remains unresolved. The direction of association remains unresolved because the same dietary and clinical factors can independently influence both microbiota composition and gastrointestinal symptoms.

Because gastrointestinal symptoms directly affect physical comfort, nutritional adherence, social participation, and family routines, they represent an important patient-centred outcome for future microbiota-focused studies. Future clinical trials should therefore incorporate validated gastrointestinal symptom assessments alongside microbiome analyses.

#### 5.3.4 The gut–brain axis and neurobehavioural outcomes

Neurocognitive impairment, behavioural difficulties, anxiety, and psychosocial challenges are important determinants of HRQoL in several paediatric IMDs, particularly disorders associated with toxic metabolite accumulation, such as PKU and organic acidemias (Bösch et al., 2021; Zeltner et al., 2014). The microbiota–gut–brain axis has attracted increasing attention as a possible pathway linking intestinal microbial activity with neurological function through immune, endocrine, metabolic, and neural mechanisms (Cryan et al., 2019; Strandwitz, 2018). Experimental studies suggest that microbial metabolites may influence neurotransmitter pathways, neuroinflammation, and stress responses (Thomas et al., 2009; Wahlström et al., 2016). Reduced SCFA production and altered microbial signalling have been associated with behavioural phenotypes in experimental models; however, translation of these findings to paediatric IMDs remains uncertain. In metabolic disorders, neurological manifestations are often strongly influenced by disease-specific factors, including toxic metabolite accumulation, metabolic instability, and genetic mechanisms. Therefore, microbiota-mediated effects should currently be considered as potentially modifying factors rather than established drivers of neurodevelopmental outcomes.

#### 5.3.5 Current evidence linking microbiota-directed interventions and QoL

Microbiota-directed interventions represent an emerging therapeutic concept that may eventually complement established IMD treatments. Potential approaches include dietary fibre optimisation, prebiotic supplementation, probiotics, synbiotics, engineered microbial strains, and other microbiome-modulating strategies. Small exploratory studies have suggested that modifying microbial composition may influence gastrointestinal symptoms or microbial diversity in selected IMDs, particularly PKU and organic acidemias (Bordugo et al., 2021; Erny et al., 2015). However, robust evidence demonstrating improvement in HRQoL following microbiota-directed therapy is currently lacking. Future intervention studies should move beyond microbiological endpoints alone and evaluate clinically meaningful outcomes, including metabolic control, gastrointestinal symptom burden, neuropsychological function, caregiver impact, and validated HRQoL measures. A patient-centred framework integrating microbiome profiling with PROMs will be essential to determine whether microbiota modulation can provide meaningful benefit for children living with IMDs (Figure 1). No robust paediatric IMD trial has demonstrated that microbiota-directed treatment improves HRQoL. Evidence for probiotics, prebiotics, synbiotics, FMT, engineered microbes, and phage-based approaches is therefore discussed according to the highest level of evidence currently available rather than assumed clinical efficacy.

**Figure 1:**
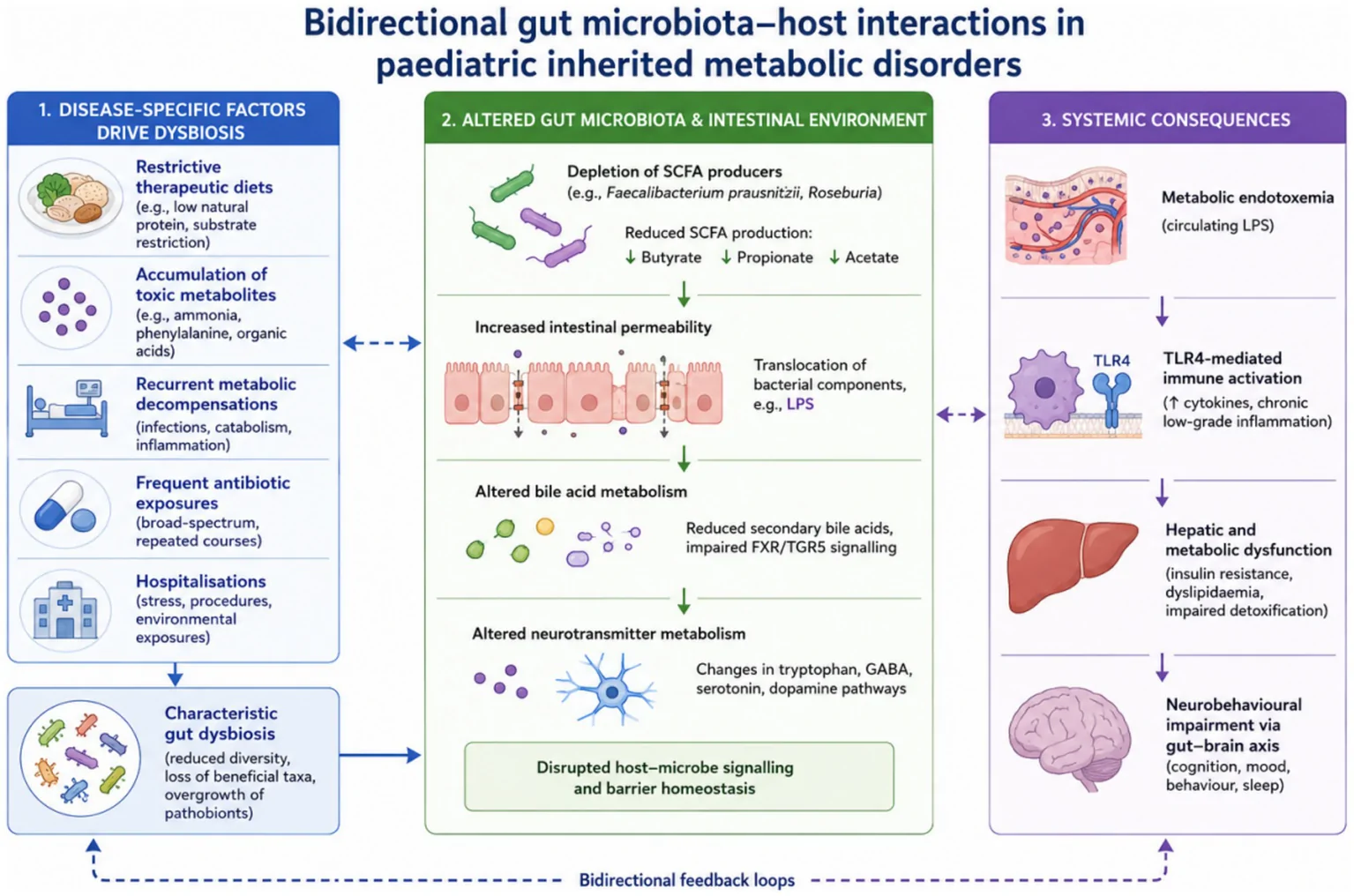
Bidirectional gut microbiota–host interactions in paediatric inherited metabolic disorders. Left panel: Disease-specific factors, including restrictive therapeutic diets, accumulation of toxic metabolites, recurrent metabolic decompensations, frequent antibiotic exposures, and hospitalisations, drive characteristic dysbiosis. Centre panel: Depletion of short-chain fatty acid (SCFA) producers (e.g., *Faecalibacterium prausnitzii, Roseburia*) reduces butyrate, propionate, and acetate availability; increased intestinal permeability enables lipopolysaccharide (LPS) translocation; altered bile acid and neurotransmitter metabolism disrupts host signalling. Right panel: Systemic consequences include metabolic endotoxaemia, Toll-like receptor 4 (TLR4)-mediated immune activation, hepatic and metabolic dysfunction, and neurobehavioural impairment via the gut–brain axis. Dashed arrows indicate bidirectional feedback loops. SCFA, short-chain fatty acid; LPS, lipopolysaccharide; TLR4, Toll-like receptor 4; 5-HT, serotonin; GABA, γ-aminobutyric acid.

Current evidence supports the concept that gut microbiota may represent one component of the complex biological network influencing symptoms and well-being in paediatric IMDs. Nevertheless, the relationship between microbiota alterations and HRQoL remains largely hypothetical, with insufficient direct clinical evidence to establish causality.

The major determinants of HRQoL in IMDs remain disease severity, metabolic control, treatment burden, gastrointestinal symptoms, neurodevelopmental outcomes, and family factors. Future longitudinal studies integrating microbiome profiling, metabolomics, and patient-reported outcomes will be required to determine whether microbiota-related pathways represent clinically meaningful targets for improving quality of life.

## 6 Microbiota-directed interventions

### 6.1 Rationale and dietary modulation

Growing recognition of host–microbiome interactions has garnered interest in therapeutic approaches that modify gut microbial composition or function (Table 4). Because many IMDs are characterised by restrictive dietary therapies, altered nutrient availability, gastrointestinal dysfunction, and potential microbial dysbiosis, microbiota-targeted interventions have been proposed as adjunctive strategies to improve metabolic control, gastrointestinal symptoms, and quality of life (Kirby et al., 2019; Mancilla et al., 2021). However, enthusiasm must be balanced against the limited quantity and quality of available evidence. Most studies in paediatric IMDs are small, observational, or exploratory, while many proposed interventions are supported primarily by evidence from obesity, inflammatory bowel disease, and other chronic conditions rather than IMDs themselves (Hill et al., 2014; Sanders et al., 2019). Accordingly, evidence from obesity, inflammatory bowel disease, or other common conditions is used only to explain biological rationale and is not treated as evidence of efficacy in paediatric IMDs.

**Table 4:** Microbiota-directed interventions in paediatric inherited metabolic disorders: current evidence and future directions.

Intervention type	Mechanism	Evidence in IMDs	Evidence source population	Potential benefits	Safety considerations
Precision Dietary Modification (Fibre Optimisation)	Increases fermentation substrate; stimulates butyrate producers; enhances tight junction expression and gut barrier integrity	Observational studies in PKU and GSD show fibre-microbiota diversity correlation; no RCTs have been completed	PKU, GSD; extrapolated from non-IMD metabolic and IBD cohorts	Improved constipation and gut motility; enhanced SCFA production; possible reduced systemic inflammation; better metabolic stability in GSD	Risk of excessive Phe/protein intake in PKU/organic acidemias if natural fibre sources are not carefully selected; potential carbohydrate overload in GSD
Prebiotic Supplementation (GOS, FOS, Resistant Starch)	Selective fermentation by beneficial commensals (*Bifidobacterium, Lactobacillus, F. prausnitzii*); increased SCFA production; pH reduction inhibiting pathobionts	Case series in PKU suggest improved stool frequency; preclinical GSD data show enhanced butyrate	PKU case series; preclinical GSD models; extrapolated from healthy infant and adult IBD trials	Restoration of depleted SCFA producers; improved gut barrier function; possible immune modulation	Bloating and abdominal discomfort from excessive fermentation; gas production may aggravate feeding intolerance in organic acidemias; osmotic diarrhoea risk
Probiotic Supplementation (Bifidobacterium, Lactobacillus)	Competitive exclusion of pathogens; enhancement of tight junction proteins; modulation of mucosal IgA and Treg responses	Small pilot studies in PKU show modest microbial shifts; no robust clinical outcome data in organic acidemias or GSD	PKU pilot studies; extrapolated from non-IMD paediatric antibiotic-associated diarrhoea and functional GI disorder trials	Reduced antibiotic-associated diarrhoea; possible alleviation of mild GI symptoms; improved microbial diversity in stable outpatients	Bacteremia risk in immunocompromised or central-line-dependent patients; strain-specific effects require careful selection; limited data in metabolically unstable patients
Synbiotics (Probiotics + Prebiotics)	Synergistic enhancement of probiotic colonisation and metabolic activity; sustained SCFA production; multi-targeted immune and barrier modulation	No published paediatric IMD trials; extrapolated evidence from paediatric obesity suggests improved inflammatory markers	Exclusively non-IMD populations (obesity, healthy children)	Greater efficacy than single-component therapy; tailored combinations based on individual dysbiosis profiles	Formulation complexity; potential synergistic adverse effects; requires rigorous strain selection compatible with IMD metabolic constraints
Next-Generation Probiotics (Engineered/Commensal SCFA Producers)	Genetically modified or selected strains with enhanced butyrate/propionate production or Phe/BCAA metabolism capacity	Preclinical proof-of-concept only; engineered *E. coli* Phe-degrading strains tested *in vitro* and in animal models	PKU preclinical models only; no human IMD trials	Disease-specific metabolic adjunct; possible reduction in dietary strictness; restoration of deficient metabolites	Regulatory hurdles for GMOs; unknown long-term colonisation stability; theoretical risk of metabolic pathway interference
Faecal Microbiota Transplantation (FMT)	Complete restoration of donor microbial ecosystem; introduction of diverse SCFA producers, bile acid modifiers, and immune-modulating commensals	No clinical trials in paediatric IMDs; indirect safety data from *C. difficile* resolution in immunocompromised children	Non-IMD populations (adult *C. difficile*, IBD, metabolic syndrome; paediatric *C. difficile*)	Most comprehensive dysbiosis correction; potential sustained remission of GI symptoms; possible metabolic benefits via restored microbial metabolite pools	Transmission of infectious agents or antibiotic-resistant organisms; donor metabolite profile may be incompatible with IMD constraints; immune suppression concerns; ethical complexity in paediatric vulnerable populations
Phage Therapy (Targeted Pathobiont Suppression)	Bacteriophage-mediated lysis of specific pathobionts (e.g., Enterobacteriaceae) without disrupting overall community structure	Preclinical only; no paediatric metabolic disease applications published	Non-IMD preclinical models only	Highly targeted intervention; reduced off-target microbial effects; potential for personalised pathobiont elimination	Risk of phage resistance; unintended horizontal gene transfer; immune responses to phage particles; extremely limited paediatric safety data
Microbiota-Directed Complementary Foods (MDCFs)	Formulated foods containing specific glycans and proteins that selectively enrich growth-promoting taxa and enhance mucosal barrier function	None; developed primarily for undernutrition and environmental enteropathy in low-income settings	Non-IMD undernutrition trials only	Could be adapted for IMD-specific medical foods to simultaneously meet metabolic and microbial needs	Must integrate with existing protein/carbohydrate restrictions; risk of micronutrient imbalance if not carefully formulated

GOS, Galacto-oligosaccharides; FOS, Fructo-oligosaccharides; SCFA, Short-chain fatty acid; FMT, Faecal microbiota transplantation; MDCF, Microbiota-directed complementary food; GMO, Genetically modified organism; PKU, Phenylketonuria; GSD, Glycogen storage disease; IMD, Inherited metabolic disorder; Phe, Phenylalanine; BCAA, Branched-chain amino acid; GI, Gastrointestinal; RCT, Randomised controlled trial; IBD, Inflammatory bowel disease.

Diet represents the most powerful and consistently demonstrated determinant of gut microbial composition (David et al., 2014; Singh et al., 2017). Because dietary therapy constitutes the cornerstone of management for many IMDs, microbial alterations observed in these disorders may partly reflect long-term nutritional interventions. In PKU, lifelong restriction of natural protein combined with phenylalanine-free medical formulas substantially alters substrate availability for microbial fermentation (Mancilla et al., 2021; Ostrowska et al., 2024). Several investigators have suggested that optimising fibre intake within dietary restrictions may improve microbial diversity and SCFA production (MacDonald et al., 2020). Similarly, specialised dietary regimens used in organic acidemias and GSDs may influence microbial metabolic pathways through changes in carbohydrate and protein availability (Baumgartner et al., 2014; Kishnani et al., 2014). These dietary drivers initiate the mechanistic cascade depicted in Figure 2, wherein substrate restriction alters microbial composition and subsequently SCFA production. However, direct evidence demonstrating clinically meaningful microbiome-mediated benefits from dietary modification remains limited. Importantly, dietary interventions in IMDs must prioritise metabolic safety; strategies designed to alter gut microbiota should never compromise established metabolic treatment protocols. Any microbiota-oriented dietary modification must remain subordinate to established disease-specific nutritional targets and should be evaluated for metabolic safety before microbiome or HRQoL benefit is considered.

**Figure 2:**
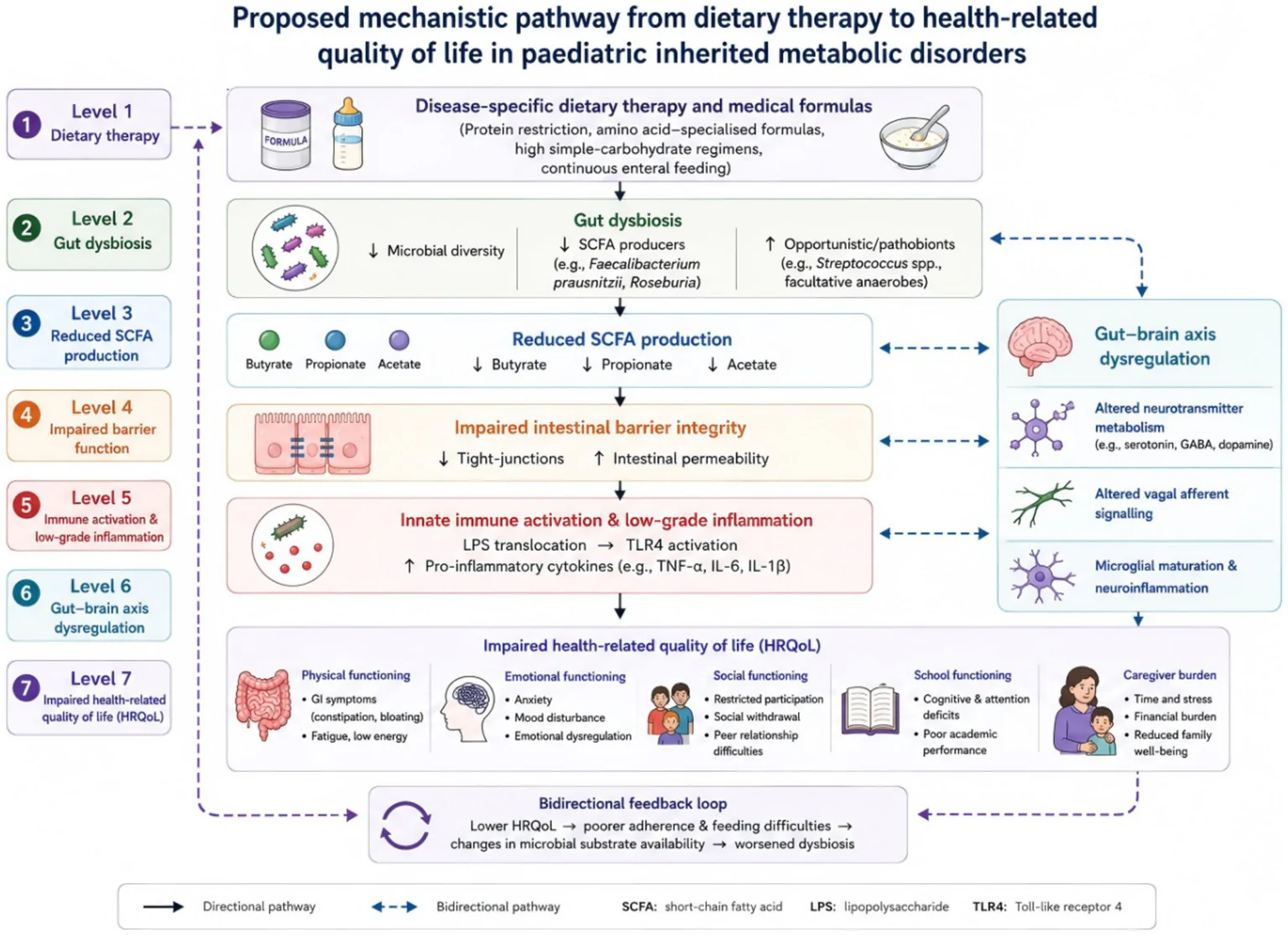
Proposed mechanistic pathway from dietary therapy to health-related quality of life in paediatric inherited metabolic disorders. Disease-specific dietary therapy and medical formulas (Level 1) may alter microbial substrate availability and contribute to gut microbiota dysbiosis (Level 2), characterized by reduced microbial diversity and depletion of SCFA-producing taxa. Reduced SCFA production (Level 3), particularly butyrate, propionate, and acetate, may impair intestinal barrier integrity (Level 4), increasing permeability and facilitating LPS translocation with TLR4-mediated innate immune activation and chronic low-grade inflammation (Level 5). In parallel, altered microbial signalling may disrupt gut–brain communication through changes in neurotransmitter metabolism, vagal afferent signalling, and microglial maturation (Level 6). These converging pathways may contribute to impaired health-related quality of life (Level 7), including gastrointestinal and physical symptoms, emotional and neurobehavioural difficulties, social and school impairment, and increased caregiver burden. Solid arrows indicate proposed directional pathways, whereas dashed arrows indicate bidirectional feedback loops. This conceptual framework is intended for hypothesis generation and does not establish causality.

### 6.2 Probiotics, prebiotics, and synbiotics

Probiotics are defined as live microorganisms that confer a health benefit when administered in adequate amounts (Hill et al., 2014; Kalliomäki et al., 2007). Proposed mechanisms include competitive exclusion of pathogenic organisms, enhancement of intestinal barrier function, modulation of immune responses, and production of beneficial metabolites. In PKU specifically, probiotic strains capable of metabolising phenylalanine or producing tyrosine have been proposed as adjunctive therapies, though clinical data remain limited to small pilot studies (Markowiak-Kopeć and Śliżewska, 2020; Shaji Thomas et al., 2021). Evidence supporting probiotic use in paediatric IMDs remains limited to small pilot studies and case reports. One notable study in propionic acidemia demonstrated measurable alterations in microbial composition following probiotic supplementation, although clinical outcomes were not definitively established (Bordugo et al., 2021).

Outside the IMD field, randomised controlled trials have demonstrated modest benefits of selected probiotic strains for antibiotic-associated diarrhoea, functional gastrointestinal disorders, and some inflammatory conditions (Hill et al., 2014; Sanders et al., 2019). Whether these findings are applicable to IMDs remains uncertain. At present, there is insufficient evidence to recommend routine probiotic administration for improving metabolic control, gastrointestinal symptoms, or HRQoL in IMDs. Furthermore, safety considerations require careful attention. Children with central venous catheters, severe immunocompromise, intestinal barrier dysfunction, or recurrent hospitalisations may theoretically face increased risks of probiotic-associated bloodstream infections, although such events remain uncommon (Doron and Snydman, 2015; Kalliomäki et al., 2007). Consequently, strain selection, patient selection, and long-term safety evaluation remain important priorities. The available evidence therefore supports neither routine probiotic use nor a generalised expectation of HRQoL benefit in paediatric IMDs.

Prebiotics, including galacto-oligosaccharides (GOS), fructo-oligosaccharides (FOS), inulin, and resistant starches, are selectively utilised substrates that promote the growth or activity of beneficial microorganisms, whereas synbiotics combine prebiotics and probiotics in a single intervention (Gibson et al., 2017; van Trijp et al., 2024). Theoretical benefits include enhancement of SCFA production, increased abundance of beneficial taxa, and improved intestinal barrier function. Such effects may be particularly relevant in disorders characterised by depletion of butyrate-producing bacteria, including PKU (Cannet et al., 2023; Mancilla et al., 2021). A recent comprehensive review highlighted the potential of synbiotic supplementation to reduce low-grade inflammation and improve metabolic markers in paediatric metabolic dysfunction, suggesting translatable benefits for IMDs characterised by inflammatory dysbiosis (Koller et al., 2025; Zierer et al., 2018). However, evidence specific to paediatric IMDs remains extremely limited. Most available data derive from healthy populations or common chronic diseases. Furthermore, tolerance of prebiotic supplementation may be challenging in patients with gastrointestinal dysmotility, bloating, or feeding intolerance. No published paediatric IMD trial currently provides sufficient evidence that prebiotics or synbiotics improve metabolic outcomes, gastrointestinal symptoms, or HRQoL; evidence from non-IMD populations should be considered indirect.

### 6.3 Faecal microbiota transplantation and engineered therapeutics

Faecal microbiota transplantation (FMT) involves the transfer of processed stool from healthy donors to recipients to restore microbial community structure and function (Cammarota et al., 2017; Yadegar et al., 2024). FMT has demonstrated substantial efficacy in recurrent *Clostridioides difficile* infection and is being investigated in inflammatory bowel disease, metabolic syndrome, and autism spectrum disorder (Cammarota et al., 2017; Conover et al., 2023; Ooijevaar et al., 2019). These successes have generated interest in potential applications for IMDs. However, safety concerns—including the risk of transmitting pathogens, introducing antibiotic-resistant organisms, or transferring metabolically active donors whose profiles may be incompatible with IMD metabolic constraints—necessitate rigorous screening and gnotobiotic validation in animal models prior to clinical application (Barati et al., 2024; Schwimmer et al., 2019). No adequately powered clinical trials have evaluated efficacy, optimal donor selection, long-term safety, or effects on metabolic outcomes in these populations. Given these uncertainties, FMT should presently be considered an experimental intervention within IMDs. No clinical trial has established FMT efficacy in paediatric IMDs, and the intervention should not currently be considered part of routine IMD care.

Advances in synthetic biology have enabled the development of genetically engineered microorganisms designed to metabolise toxic substrates, produce therapeutic compounds, or deliver biologically active molecules within the gastrointestinal tract (Barati et al., 2024; Isabella et al., 2018). One notable example is engineered *Escherichia coli Nissle* strains developed to degrade phenylalanine in PKU (Puurunen et al., 2021; Triassi et al., 2023). Preclinical studies and early-phase clinical investigations have demonstrated proof-of-concept for microbial substrate degradation and reduction of systemic phenylalanine levels (Isabella et al., 2018; Kurtz et al., 2019). Phage therapy targeting pathobionts and microbiota-directed complementary foods are additional innovative approaches under preclinical investigation, though their application in paediatric rare diseases remains theoretical (Jalili-Firoozinezhad et al., 2019; Yatsunenko et al., 2012). Unlike conventional antibiotics, phages may permit modification of microbial ecosystems while preserving broader community structure (Hsu et al., 2019). These approaches are particularly attractive because they directly target disease-specific metabolic pathways rather than broadly modifying microbial communities. However, substantial challenges remain, including long-term colonisation stability, regulatory approval pathways, biosafety considerations, host immune responses, and manufacturing scalability. Moreover, no clinical studies evaluating phage therapy in paediatric IMDs have been published. For engineered microbes, the evidence remains predominantly preclinical; claims of clinical benefit should therefore be avoided. Additional concerns include unintended metabolic activity, horizontal gene transfer, immune responses, persistence or shedding, and the regulatory requirements for genetically modified organisms.

### 6.4 Precision microbiome medicine and clinical translation

Emerging technologies are shifting the field beyond simple taxonomic characterisation towards functional analysis of host–microbiome interactions. Integration of metagenomics, metabolomics, transcriptomics, proteomics, and clinical phenotyping may facilitate the development of personalised microbiome interventions tailored to individual metabolic disorders (Franzosa et al., 2014, 2019; Zierer et al., 2018). Such approaches may help identify disease-specific microbial signatures, functional metabolic pathways, and biomarkers that predict treatment response. However, implementation remains challenging due to high costs, methodological complexity, limited standardisation, and difficulties in interpreting large, multidimensional datasets.

Overall, microbiota-directed interventions represent a promising frontier in the management of paediatric inherited metabolic disorders. However, the current evidence base remains insufficient to support routine clinical implementation beyond standard nutritional management. Future progress will require multicentre clinical trials integrating microbiome profiling, functional metabolomics, host-response biomarkers, and validated patient-reported outcome measures. Such studies will be essential to determine whether microbiota-targeted interventions can provide clinically meaningful improvements in metabolic control, gastrointestinal health, and health-related quality of life. Until such evidence is available, microbiota-directed interventions should be regarded as investigational adjuncts rather than established therapies for HRQoL improvement.

## 7 Controversies and competing perspectives

Several unresolved debates characterise the microbiome–IMD field. First, whether observed dysbiosis represents a primary disease mechanism or merely a secondary consequence of restrictive diets, antibiotics, and metabolic instability remains contested (David et al., 2014; Kirby et al., 2019). Proponents of the “diet-first” hypothesis argue that microbial alterations largely reflect substrate deprivation and would normalise if dietary restrictions were relaxed—a proposition that is ethically and clinically untestable in most IMDs (MacDonald et al., 2020). Conversely, the “microbiome-first” school contends that independent microbial pathways modulate disease severity, supported by evidence that germ-free animals colonised with IMD-associated microbiota exhibit transferable phenotypes (Alula et al., 2023; Faith et al., 2013). Resolution of this debate is critical: if dysbiosis is purely secondary, microbiota-directed therapies may offer limited value beyond dietary optimisation. At present, neither position is adequately supported by paediatric clinical evidence, and the apparent conflict may reflect the fact that disease biology and treatment exposure are closely intertwined.

A critical and underappreciated confounder is the effect of disease-specific dietary therapy on microbial composition. In PKU, lifelong reliance on phenylalanine-free medical formulas and severe restriction of natural protein intake substantially reduce dietary fibre, resistant starch, and complex carbohydrate substrates available for microbial fermentation, independently selecting for a less diverse, less butyrate-rich community (MacDonald et al., 2020; Mancilla et al., 2021). In organic acidemias, severe protein restriction and reliance on elemental amino acid mixtures limit substrate diversity, while recurrent metabolic decompensations necessitate frequent broad-spectrum antibiotic exposure, compounding dysbiosis (Baumgartner et al., 2014; Salazar et al., 2023). In GSD, high simple-carbohydrate loads from uncooked cornstarch therapy and continuous glucose delivery alter colonic pH and favour saccharolytic over cellulolytic taxa (Hannibal et al., 2020; Kishnani et al., 2014). Enteral feeding regimens—whether continuous nocturnal nasogastric or gastrostomy-based—further modify gastrointestinal transit time, gastric pH, and small intestinal bacterial exposure, representing independent microbial selectors. These factors should be treated as major potential confounders in future microbiome studies rather than secondary background variables.

Disentangling disease-specific from diet-induced microbial alterations is methodologically challenging. Future studies should therefore incorporate prospective dietary records quantifying medical formula composition, daily fibre intake, macronutrient distribution, antibiotic exposure histories, and feeding modality alongside microbiome sampling. Where ethically and practically feasible, dietary-matched control groups (e.g., healthy children on similarly restrictive diets for non-metabolic indications) would help isolate disease-specific microbial signatures. In the absence of such controls, stratified analyses by formula type, fibre intake tertile, and antibiotic exposure status should be considered minimum standards for observational IMD microbiome research. Prospective recording of dietary and treatment exposures should therefore be considered essential for interpreting disease-specific microbial signatures.

Second, divergent views exist regarding the relative importance of taxonomic composition versus functional output. Traditional 16S rRNA studies have dominated the IMD literature, yet metagenomic and metabolomic analyses suggest that taxonomic profiles may poorly predict microbial function (Franzosa et al., 2014; Zierer et al., 2018). This methodological schism has practical implications: taxonomic data may guide probiotic selection, whereas functional data are required for precision dietary or engineered interventions.

Third, the therapeutic risk–benefit calculus for microbiota manipulation in vulnerable paediatric populations remains controversial. While some investigators advocate proactive probiotic or prebiotic supplementation to restore SCFA production (Hill et al., 2014; Sanders et al., 2019), others caution that introducing live microorganisms into immunocompromised or metabolically unstable children carries underquantified risks, including bacteraemia and metabolic pathway interference (Barati et al., 2024; Doron and Snydman, 2015). Similarly, enthusiasm for FMT must be balanced against concerns about donor metabolite incompatibility and pathogen transmission in already fragile patients (Cammarota et al., 2017; Yadegar et al., 2024). In paediatric IMDs, this risk assessment must be individualised because metabolic instability, central venous access, intestinal dysfunction, and immune vulnerability may alter the balance between potential benefit and harm.

Finally, the integration of patient-reported outcomes into microbiome research has generated debate. Traditional metabolic disease research prioritises biochemical control as the primary endpoint, whereas patient-centred frameworks argue that HRQoL, caregiver burden, and functional status are equally valid targets (Bösch et al., 2021; Pascoal et al., 2018). Whether microbiota-directed interventions can realistically improve these broader outcomes—given the multifactorial determinants of quality of life in IMDs—remains a point of active discussion. Because HRQoL is multifactorial, any future intervention trial should prespecify clinically meaningful patient-reported outcomes rather than infer benefit from microbial changes alone.

## 8 Limitations, emerging technologies, and future directions

### 8.1 Limitations of current evidence

Although interest in host–microbiome interactions in IMDs has expanded, evidence remains limited. First, most paediatric IMD studies involve small sample sizes. Disease rarity restricts recruitment to single-centre cohorts of fewer than 50 participants (Mancilla et al., 2021; Ostrowska et al., 2024; Pinheiro de Oliveira et al., 2016), reducing statistical power. Second, substantial heterogeneity exists across IMDs. Conditions such as PKU, MMA, PA, GSD, LSD, and mitochondrial diseases differ markedly in pathophysiology (Ferreira et al., 2021), so findings cannot be generalised. Third, most investigations employ cross-sectional designs that identify associations but cannot establish causality (Knight et al., 2018). Observed alterations may reflect disease contributors or treatment consequences. Fourth, methodological variability in stool collection, DNA extraction, sequencing platforms, and bioinformatic analyses complicates comparisons (Costea et al., 2017; Sinha et al., 2017). Standardisation remains problematic in rare diseases. Fifth, most studies focus on taxonomic composition rather than function. Taxonomic profiling may not reflect metabolic activity or host–microbiome interactions (Fan and Pedersen, 2021; Franzosa et al., 2019; Stanislawski et al., 2018; Zierer et al., 2018). Functional multi-omics remain uncommon in paediatric IMDs. Sixth, dietary therapy confounds microbiota signatures, as nutritional management profoundly influences composition (David et al., 2014; Krautkramer et al., 2016; Melo et al., 2019; Singh et al., 2017). Distinguishing disease-specific from diet-induced alterations remains challenging (Mancilla et al., 2021; Ostrowska et al., 2024; Pinheiro de Oliveira et al., 2016). Restrictive therapeutic diets, reduced dietary fibre intake, specialised medical formulas, recurrent metabolic decompensations, and antibiotic exposure may all influence microbial composition and function, yet these factors are infrequently controlled for in observational studies. Without diet-matched controls or detailed nutritional covariate adjustment, claims regarding disease-specific dysbiosis must be interpreted cautiously. Seventh, and most critically, intervention evidence is virtually absent. Most probiotic, prebiotic, and dietary studies are small or exploratory (Sanders et al., 2019), with robust randomised trials largely absent. No study has simultaneously incorporated patient-reported outcomes or quality of life measures alongside microbiota-directed therapy in paediatric IMDs (Bösch et al., 2021; Pascoal et al., 2018). This limitation must temper any clinical enthusiasm for microbiome modification in these populations. Eighth, direct evidence linking microbiome characteristics to HRQoL is particularly limited: most studies evaluate these domains separately, and few have concurrent microbiome, metabolomic, clinical, and validated PROM data. This prevents reliable attribution of HRQoL differences to microbiota alterations.

### 8.2 Emerging technologies

The integration of 16S rRNA sequencing, shotgun metagenomics, metabolomics, and transcriptomics enables comprehensive characterisation of host–microbiota interactions in IMDs (Franzosa et al., 2014; Zierer et al., 2018). Metabolomic profiling can distinguish disease-specific microbial signatures from host-derived metabolite alterations, while metatranscriptomics reveals functional pathways active *in vivo* rather than merely taxonomic potential (Cani et al., 2008; Pasolli et al., 2016; Song et al., 2017) (Figure 3). Machine learning algorithms applied to microbiota compositional data can predict metabolic disease risk, classify disease subtypes, and identify therapeutic responders (Fan and Pedersen, 2021; Jacobs et al., 2023; Knight et al., 2018). In paediatric metabolic dysfunction-associated steatotic liver disease, machine-learning models have achieved high discriminatory accuracy between disease stages based on microbial taxa (Colmenarejo, 2020; Jacobs et al., 2023; Louca, 2025). Similar approaches could stratify IMD patients by dysbiosis severity to guide personalised interventions, though validation in rare diseases is lacking. These approaches may improve biological resolution, but predictive performance in non-IMD cohorts should not be interpreted as validated diagnostic or therapeutic performance in rare paediatric IMDs.

**Figure 3:**
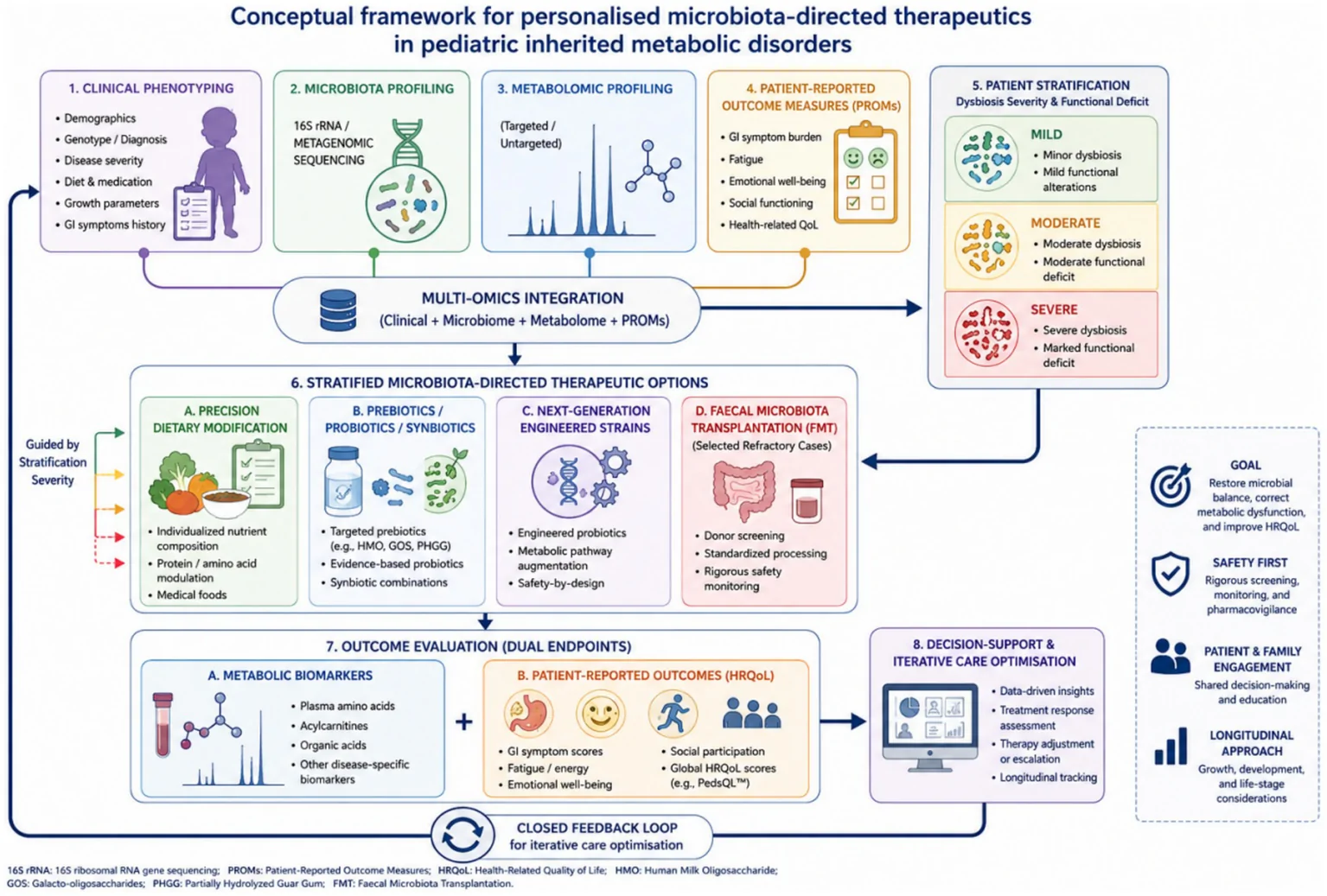
Conceptual framework for personalised microbiota-directed therapeutics in paediatric inherited metabolic disorders. The model integrates clinical phenotyping, 16S rRNA/metagenomic sequencing, metabolomic profiling, and validated patient-reported outcome measures (PROMs) into a unified decision-support system. Multi-omics data enable stratification of patients by dysbiosis severity and functional deficit. Stratified therapeutic options include precision dietary modification, prebiotics/probiotics/synbiotics, next-generation engineered strains, or, in selected refractory cases, faecal microbiota transplantation. Outcomes are evaluated by dual endpoints: metabolic biomarkers (e.g., plasma amino acids, acylcarnitines) and patient-reported health-related quality of life (HRQoL) metrics, creating a closed feedback loop for iterative care optimisation.

Gnotobiotic animal models colonised with human IMD-associated microbiota enable causal mechanistic studies impossible in human subjects (Jalili-Firoozinezhad et al., 2019; Moysidou and Owens, 2021; Viggiano et al., 2015). Organ-on-chip systems incorporating intestinal epithelium, immune cells, and microbial communities offer high-throughput platforms for testing microbiota-directed therapies under controlled conditions that mimic human physiology (Moradian et al., 2024; Puschhof et al., 2021; Yatsunenko et al., 2012). These technologies will be critical for the preclinical validation of next-generation probiotics and engineered strains prior to paediatric trials. Before translation to children, these platforms should establish reproducibility, metabolic safety, microbial containment, and disease-specific efficacy in appropriately controlled preclinical models.

### 8.3 Future research priorities

Addressing current limitations will require coordinated multidisciplinary research efforts and international collaboration. One important priority is the establishment of multicentre longitudinal cohorts. Given the rarity of many IMDs, collaborative networks will be essential to recruit sufficiently large patient populations and improve statistical power (Ferreira et al., 2021). Longitudinal designs would allow investigators to evaluate temporal changes in microbial composition, identify predictors of clinical outcomes, and better assess causal relationships. Future studies should move beyond descriptive taxonomic analyses towards integrated multi-omics approaches. Combining metagenomics, metabolomics, transcriptomics, proteomics, and host genomic data may provide a more comprehensive understanding of functional host–microbiome interactions and identify disease-specific biological pathways (Flint et al., 2012; Franzosa et al., 2019; van Karnebeek, 2018). Another key priority involves the development of standardised protocols for microbiome studies in rare diseases. Harmonisation of sampling procedures, sequencing methodologies, metadata collection, and bioinformatic pipelines would improve reproducibility and facilitate comparison across studies (Costea et al., 2017). Where possible, cohorts should begin before major treatment changes and repeatedly capture disease severity, metabolic control, age/puberty, medical formula composition, fibre and macronutrient intake, antibiotics, medications, feeding modality, and major psychosocial determinants. These variables should be incorporated into prespecified causal models rather than treated as *post hoc* covariates.

Greater emphasis should also be placed on patient-centred outcomes. Future investigations should routinely incorporate validated HRQoL instruments, PROMs, neuropsychological assessments, and caregiver-reported measures alongside microbiome analyses (Pascoal et al., 2018). Such integration would help determine whether microbiota alterations have clinically meaningful implications for daily functioning and well-being. We explicitly prioritise HRQoL-integrated microbiome cohort studies as the first future research recommendation: such studies should collect concurrent microbiome profiling (16S rRNA or metagenomics), metabolomics (SCFA quantification), intestinal permeability biomarkers (zonulin, claudin-3), and validated PROMs at multiple timepoints. Interventional research represents another critical need. Carefully designed randomised controlled trials evaluating dietary interventions, prebiotics, probiotics, synbiotics, and emerging microbiota-directed therapies are required to determine efficacy, safety, and durability of treatment effects. Clinical outcomes should extend beyond microbial composition and include metabolic control, symptom burden, neurocognitive function, healthcare utilisation, and quality of life. Furthermore, advances in synthetic biology and precision medicine may facilitate the development of engineered microbial therapeutics tailored to specific metabolic disorders (Isabella et al., 2018; Kurtz et al., 2019). Although these technologies remain investigational, they represent promising future directions that warrant careful evaluation through rigorous translational research. Qualitative research is also needed to understand patient and caregiver perspectives on microbiota-directed therapies, recognising that acceptability and adherence are critical determinants of real-world HRQoL impact. The primary objective of such studies should be to test whether microbiota-associated changes correlate temporally with clinically meaningful HRQoL trajectories, while recognising that correlation alone will not establish causality. Randomised trials should be considered only after adequate safety and feasibility data are available.

## 9 Discussion and synthesis

### 9.1 Integrating the evidence: the gut microbiota–metabolism–quality of life axis

The evidence synthesised in this review supports the conceptualisation of a gut microbiota–metabolism–quality of life axis in paediatric IMDs, but does not establish the axis as a causal pathway. As summarised in Table 1, the strongest evidence exists for PKU, where consistent alterations in microbial diversity and butyrate-producing taxa have been reported across multiple controlled observational studies. For other IMDs—organic acidemias, GSDs, LSDs, and mitochondrial disorders—evidence ranges from sparse to absent. Figure 2 provides a schematic representation of the proposed pathway from dietary therapy through microbial alterations, barrier dysfunction, immune activation, gut–brain axis disruption, and ultimate impact on HRQoL across physical, emotional, social, and school functioning domains. Nevertheless, biological plausibility is supported by mechanistic studies in non-IMD populations demonstrating that microbial metabolites influence intestinal barrier function, immune regulation, and neurobehavioural outcomes through well-characterised pathways (Gasaly et al., 2021; Ghosh et al., 2021; Scott et al., 2020). The framework should therefore be interpreted as a structured hypothesis that integrates direct PKU observations with lower-certainty evidence and mechanistic extrapolation from other populations.

The clinical relevance of these findings lies in their potential to inform new therapeutic approaches. If dysbiosis contributes to gastrointestinal symptoms, metabolic instability, or neurobehavioural impairment, microbiota-directed interventions could theoretically complement existing disease-modifying treatments, but this proposition remains untested in adequately powered paediatric IMD trials. The conceptual framework presented here—integrating microbial metabolites, barrier function, immune signalling, and patient-reported outcomes—provides a structure for hypothesis generation and study design. However, enthusiasm must be tempered by recognition of substantial evidence gaps.

### 9.2 Critical unresolved questions

Several critical questions remain unresolved. First, do microbiota alterations represent a cause or consequence of metabolic disease and its treatment? Longitudinal studies beginning before treatment initiation are needed to address causality. Second, what is the relative contribution of disease-specific pathophysiology versus therapeutic dietary interventions to observed dysbiosis? Controlled studies with appropriately matched dietary controls are needed. Third, do microbiota-directed interventions produce clinically meaningful improvements in patient-centred outcomes? Robust clinical trials integrating microbiome and patient-reported outcome measures are required. Fourth, which patients are most likely to benefit from microbiota-directed therapies? Biomarker development and patient stratification will be essential for translation. A further priority is to determine whether observed microbial signatures persist after accounting for diet, medical formula, antibiotics, feeding modality, age, disease severity, and metabolic control.

### 9.3 Implications for clinical practice

Given the current evidence limitations, no clinical recommendations can be made for routine microbiota-directed interventions in paediatric IMDs. Clinicians should continue to prioritise established disease-specific treatments while being aware of the potential influence of dietary therapies on gut microbial composition. For families interested in probiotic or prebiotic supplementation, clinicians should counsel that evidence for benefit in IMDs is currently insufficient and that theoretical safety concerns exist, particularly in metabolically unstable patients or those with central venous catheters. Probiotic, prebiotic, synbiotic, FMT, engineered microbial, or phage-based interventions should be undertaken in research settings with appropriate eligibility criteria, monitoring, and predefined safety outcomes until paediatric IMD-specific efficacy and safety are established.

### 9.4 Methodological recommendations

To advance the field, we recommend the following methodological approaches. First, standardise microbiome sampling and analysis protocols to enable cross-study comparison. Second, incorporate metabolomic and functional assessments alongside taxonomic profiling. Third, employ longitudinal designs with repeated measures to capture dynamic changes. Fourth, integrate PROMs and HRQoL instruments as primary or secondary outcomes. Fifth, utilise animal models and organ-on-chip systems to establish causality. Sixth, establish international collaborative consortia to overcome sample size limitations inherent to rare diseases. Where feasible, future studies should also use causal-inference frameworks, repeated measures, disease-specific matched controls, and preregistered outcome definitions to distinguish temporal association from causation.

## 10 Conclusion

Gut microbiota dysbiosis is an emerging but increasingly recognised feature of rare paediatric inherited metabolic disorders, with the strongest current evidence in PKU. Characteristic alterations in microbial composition—particularly depletion of SCFA-producing taxa—may be associated with differences in gut barrier function, immune signalling, and neurobehavioural phenotypes through complex host–microbiota crosstalk, but direct causal evidence in paediatric IMDs remains absent. These pathophysiological changes may intersect with quality of life, as GI symptoms, neuropsychiatric comorbidities, and treatment burden represent primary determinants of HRQoL impairment in affected children and their families. Microbiota-directed interventions, ranging from precision dietary modifications to next-generation probiotics and synbiotics, represent investigational strategies rather than established treatments for paediatric IMDs or HRQoL impairment. However, rigorous clinical trials integrating multi-omics profiling with validated patient-reported outcome measures are essential to establish efficacy, safety, and patient acceptability. As the field advances, a patient-centred framework that prioritises both metabolic control and quality of life will be critical to translating microbiota science into meaningful clinical benefit for children with rare metabolic diseases. The principal contribution of this review is therefore conceptual: it highlights a clinically relevant, testable interface between microbial biology and patient-centred outcomes while clearly identifying the substantial evidentiary gaps.
