# Supplementary material for: Gut microbiota and health-related quality of life in paediatric inherited metabolic disorders: a narrative review and conceptual framework
Source: Front Microbiol. 2026 Sep 28;17:1928657. doi: 10.3389/fmicb.2026.1928657 (PMC13655685; doi:10.3389/fmicb.2026.1928657)
Supplement: Supplementary file 1 [file Data_Sheet_1.docx]

**Supplementary Figure S1. Simplified literature search and study selection flow diagram.** Electronic searches in PubMed, Embase, and Web of Science from database inception to February 2026 retrieved approximately 3,400 records. After automated and manual deduplication, ~2,850 unique records underwent title and abstract screening. Of these, 215 full-text articles were assessed for eligibility, and 108 studies were ultimately included in the narrative synthesis based on their contribution to: (a) gut microbiota composition or function in paediatric IMDs; (b) validated HRQoL or patient-reported outcome assessment in paediatric IMDs; or (c) mechanistic pathways with explicit IMD relevance. Reasons for exclusion at full-text stage are detailed in the side panel. This diagram is provided for transparency; the review did not claim PRISMA-compliant systematic screening.
